# Outcomes of lung transplantation using TorEx ex-vivo lung perfusion

**DOI:** 10.1016/j.jhlto.2026.100615

**Published:** 2026-06-26

**Authors:** Haruchika Yamamoto, Aadil Ali, Gabriel Siebiger, Elliot Wakeam, Laura Donahoe, Jonathan C. Yeung, Andrew Pierre, Marc de Perrot, Kazuhiro Yasufuku, Thomas K. Waddell, Shaf Keshavjee, Marcelo Cypel

**Affiliations:** a Toronto Lung Transplant Program, Toronto General Hospital, University Health Network, University of Toronto, Ontario, Canada; b Department of General Thoracic Surgery, Okayama University Hospital, Okayama, Japan

**Keywords:** Ex-vivo lung perfusion, Lung transplantation, TorEX, Next-generation EVLP, Toronto EVLP system, Outcome

## Abstract

**Purpose:**

Ex-vivo lung perfusion (EVLP) has expanded donor lung utilization, with over 1000 cases performed at our center since 2008. A next-generation Toronto EVLP system (TorEx EVLP) was introduced in December 2022, enabling simplified circuit setup by loading an organ chamber into an all‑in‑one device, potentially facilitating EVLP use at less experienced centers. This study reports the initial experience with TorEx EVLP and compares outcomes with (1) first‑generation EVLP and (2) conventional lung transplantation without EVLP.

**Methods:**

In this single-center retrospective cohort study, 1067 EVLP procedures were performed from September 2008 to September 2024 (first-generation EVLP [G1], n=874; TorEx EVLP [G2], n=193). Post-transplant short- and long-term outcomes were compared among G1, G2, and contemporaneous direct transplants without EVLP (direct, n=1575).

**Results:**

Of 1067 EVLP cases, 684 were accepted for transplant (utilization: G1 64.4%, G2 62.7%), and 700 transplants were performed (including 16 split singles). G1 and G2 had significantly higher DCD donor use (direct vs G1 vs G2: 17% vs 48% vs 60%, p<0.001) and lower donor P/F ratios (422 vs 368 vs 368 mmHg, p<0.001). Recipients in G1 and G2 were older than direct recipients (59 vs 61 vs 65 years, p<0.05). Post‑transplant outcomes were similar across groups, including PGD3 at 72 h (15% vs 16% vs 10%, p=0.21), extubation within 72 h (65% vs 64% vs 68%, p=0.67), overall survival, and CLAD‑free survival.

**Conclusions:**

Despite higher-risk donor use, outcomes with both first- and next-generation Toronto EVLP systems were comparable to direct transplantation.

## Introduction

Lung transplantation is an established treatment option for patients with advanced lung disease. However, the availability of suitable donor lungs remains limited, posing challenges in organ allocation and prolonging waiting times for recipients. Efforts have been made to optimize donor lung utilization, including the development of ex vivo lung perfusion (EVLP),^1^ which facilitates the assessment and potential reconditioning of marginal donor lungs prior to transplantation. Since its implementation, EVLP has been integrated into clinical practice, contributing to an increase in available donor lungs.^2^

The Toronto EVLP method has been widely utilized to evaluate and preserve donor lungs for transplantation. Studies on conventional EVLP have reported post-transplant outcomes comparable to direct lung transplantation, despite the inclusion of extended-criteria donor lungs.^2^, ^3^ This has supported the broader use of EVLP in clinical settings. However, the procedure requires technical expertise and careful setup, which may limit accessibility in centers with less experience.

**Figure 1:**
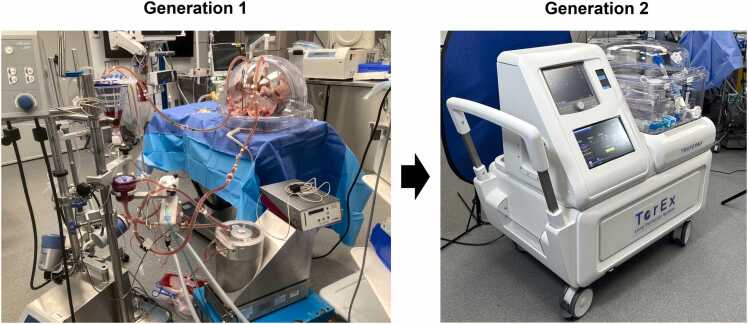
Comparison of first-generation and next-generation EVLP systems. Illustration of the conventional first-generation EVLP system (left) and the next-generation Toronto EVLP system (TorEx EVLP; right). The TorEx EVLP features a compact, all-in-one cassette that integrates the ventilator, pump, and perfusion circuit, streamlining setup and reducing personnel demands compared to the modular configuration of the first-generation system.

The next-generation Toronto EVLP system (TorEx EVLP) was designed to streamline the process, integrating an all-in-one cassette system that simplifies setup and reduces the personnel required for EVLP (Figure 1). While this system has potential advantages in procedural efficiency, its clinical effectiveness and impact on recipient outcomes compared to conventional EVLP and direct transplantation require further investigation.

This study aims to evaluate the clinical outcomes of lung transplantation using the TorEx EVLP system and compare these outcomes with those achieved using conventional EVLP and with those achieved with direct lung transplantation.

## Methods

### Study design

This study was a single-center retrospective cohort study conducted at Toronto General Hospital. The study aimed to compare post-transplant outcomes among three groups: first-generation EVLP (G1), next-generation EVLP (TorEx, G2), and conventional lung transplant recipients without EVLP (Direct to Tx). All patients who underwent lung transplantation at Toronto General Hospital between September 2008 and September 2024 were included in this study. No specific exclusion criteria were applied, ensuring comprehensive data inclusion. Donor lungs classified as marginal were subjected to EVLP assessment to evaluate transplant suitability. Lungs deemed unsuitable following EVLP assessment were documented for utilization analysis but were not included in post-transplant outcome evaluations.

### Donor lung characteristics

The following donor lung parameters were analyzed among the three groups: history of cigarette smoking, donor subtype (Donation after Brain Death; DBD/ Donation after Circulatory Death; DCD), age, sex, body mass index (BMI), cause of death, partial pressure of oxygen (PO2) / fractional inspired O2 concentration (FiO2) ratio (P/F ratio), and retrieval logistics: donor organ retrieval performed by an external retrieval team.

In addition, the cold ischemia time 1 (CIT1; time from flushing at procurement to the start of EVLP in EVLP cases, or from flushing at procurement to reperfusion after transplantation in non-EVLP cases), cold ischemia time 2 (CIT2; time from the end of EVLP to reperfusion after lung transplantation in EVLP cases), warm ischemia time (WIT; time for lung implantation. For bilateral lung transplants, WIT was measured for the initially implanted side), EVLP perfusion time, and total ischemia time (TIT; corresponding to CIT1+CIT2+WIT) were compared. Post-EVLP utilization rates were also compared based on donor subtype.

This study was conducted in accordance with the ethical guidelines of the Toronto General Hospital Research Ethics Board and received formal approval from the institution’s ethics committee (REB25–5021).

### Recipient characteristics

Recipient clinical characteristics were compared across study groups to evaluate their impact on post-transplant outcomes. The parameters analyzed included age, sex, BMI, primary lung disease diagnosis, pre-transplant extracorporeal membrane oxygenation (ECMO) support, and transplant type (single vs. bilateral lung transplantation).

### Post-transplant outcomes

Post-transplant outcomes were evaluated based on predefined study endpoints to determine the impact of preservation strategies on recipient outcomes. The primary endpoint was the incidence of International Society for Heart and Lung Transplantation (ISHLT) Grade 3 Primary Graft Dysfunction (PGD) at 72 h post-transplantation.^4^ Secondary endpoints included time to extubation, duration of mechanical ventilation (≧72 h vs. <72 h), requirement for post-transplant ECMO, ICU length of stay, hospital length of stay, overall survival, and chronic lung allograft dysfunction (CLAD)-free survival.

### Statistical analysis

Donor and recipient characteristics were summarized using descriptive statistics, with continuous variables reported as medians and interquartile ranges (IQR) and categorical variables presented as frequencies and percentages. For comparisons among the three study groups, Kruskal-Wallis tests were performed for continuous variables, while Chi-squared tests was used for categorical variables. In pairwise comparisons between two groups, Bonferroni correction for multiple testing were applied for categorical and continuous variables. Survival analyses, including overall survival and CLAD-free survival, were conducted using the Kaplan-Meier method, with inter-group differences assessed via log-rank tests. Censoring events included death, loss to follow-up, or completion of the study period. Missing values were not replaced. Statistical significance was defined as p < 0.05. All statistical analyses were performed using EZR version 1.40 (Saitama Medical Center, Jichi Medical University, Saitama, Japan) [23]. GraphPad Prism 7.04 software program (San Diego, CA, USA) was used for graph editing.

## Results

**Figure 2:**
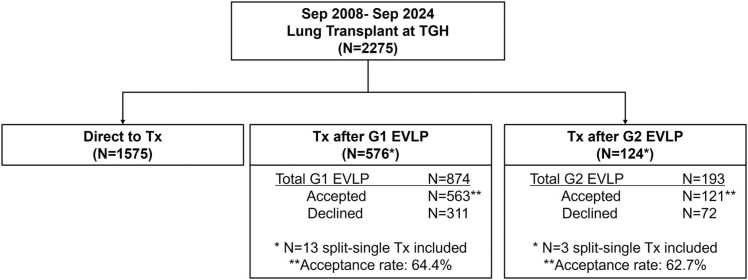
Study Flowchart. Flow diagram illustrating patient inclusion and group allocation. Between September 2008 and September 2024, a total of 2275 lung transplant recipients were categorized into three groups: Direct transplantation (n = 1575), first-generation EVLP (G1, n = 576), and next-generation EVLP using the TorEx system (G2, n = 124). Donor lungs assessed but not transplanted were excluded from outcome analysis.

The study flowchart is shown in Figure 2. Between September 2008 and September 2024, a total of 2275 patients underwent lung transplantation at Toronto General Hospital. Among them, 1575 patients received conventional lung transplantation without EVLP (Direct to Tx), 576 patients received lungs preserved using first-generation EVLP (G1), and 124 patients underwent transplantation following assessment with the next-generation EVLP system (TorEx, G2). The acceptance rate for donor lungs subjected to EVLP was 64.4% in G1 and 62.7% in G2 (p = 0.68).

Donor characteristics are summarized in Table 1. Donor age was significantly higher in G2 compared to G1 (p = 0.01), while no significant difference was observed between G2 and Direct (p = 0.98). The distribution of donor type varied across groups, with a significantly higher proportion of DCD donors in both G1 (48.3%) and G2 (60.5%) compared to Direct (17.1%, p < 0.001). Additionally, donor P/F ratio was significantly lower in EVLP groups compared to Direct (median G1 vs G2 vs Direct to Tx 368 vs 367.5 vs. 422, p < 0.001). Furthermore, the proportion of non-UHN retrieval cases was significantly higher in the EVLP groups, with 6.9% in G1 and 16.1% in G2, compared to 2.9% in Direct to Tx (p < 0.001).

**Table 1:** Comparison of Donor Lung Characteristics Across Study Groups

**Factor**		**Direct to Tx**	**EVLP (G1)**	**EVLP (G2)**	p-value			
		**(N=1575)**	**(N=576)**	**(N=124)**	**Direct vs G1 vs G2**	**Direct vs G1**	**Direct vs G2**	**G1 vs G2**
Donor Cigarette Use	Yes	757 (49.8%)	338 (60.7%)	70 (57.4%)	<0.001	<0.001	0.39	1
Donor Type	DBD	1306 (82.9%)	298 (51.7%)	49 (39.5%)	<0.001	<0.001	<0.001	0.053
	DCD	269 (17.1%)	278 (48.3%)	75 (60.5%)				
Donor age		50 (IQR 34–61)	47 (IQR 32–57)	52 (IQR 38.5–61)	<0.001	0.0014	0.98	0.01
Donor sex	Female	748 (47.5%)	213 (37%)	46 (37.1%)	<0.001	<0.001	0.09	1
	Male	827 (52.5%)	363 (63%)	78 (62.9%)				
Dnor BMI		25.8 (IQR 22.7–29.6)	26.7 (IQR 23.8–31.1)	27.6 (IQR 23.8–31.6)	<0.001	<0.001	0.009	1
Donor cause of death	Anoxia/Cardiac Arrest	395 (25.1%)	233 (40.5%)	40 (32.3%)	<0.001	<0.001	<0.001	<0.001
	Cerebrovascular/Stroke	740 (47%)	188 (32.6%)	34 (27.4%)				
	Head Trauma	336 (21.3%)	108 (18.8%)	17 (13.7%)				
	Other	104 (6.6%)	47 (8.2%)	33 (26.6%)				
Donor PO2/FiO2 ratio		422 (IQR 364–482)	368 (IQR 304–442)	367.5 (IQR 317–447)	<0.001	<0.001	<0.001	1
External retrieval team retrieval	Yes	45 (2.9%)	39 (6.9%)	20 (16.1%)	<0.001	<0.001	<0.001	0.005

Tx, transplant; G1, first-generation; G2, next-generation; DBD, donation after brain death; DCD, donation after circulatory death; IQR, interquartile range; BMI, body mass index; PO2, partial pressure of oxygen; FiO2, fractional inspired O2 concentration; UHN, University Health Network

Summary of preservation and ischemia times is provided in Table 2. Cold ischemia time (CIT1) was prolonged in G2 compared to G1, with a median of 377 min (DBD) and 445 min (DCD) in G2, versus 285 min (DBD) and 265 min (DCD) in G1 (p < 0.001). EVLP perfusion time was significantly shorter in G2 compared to G1, with a median of 217.5 min in G2 vs. 262 min in G1 (p < 0.001). Cold ischemia time following EVLP (CIT2) was also significantly longer in G2 compared to G1, with a median of 392.5 min (DBD) and 429 min (DCD) in G2, versus 242 min (DBD) and 269 min (DCD) in G1 (p < 0.001). Warm ischemia time (WIT, implantation time) remained similar across groups in DCD transplants (p = 0.171), but in DBD donors, WIT was shorter in G2 compared to G1 (median 62 min vs. 71 min, p < 0.001) and to Direct to Tx (median 69 min, p = 0.). Total ischemia time (TIT= CIT1 + CIT 2+ WIT for EVLP groups and CIT+WIT for Direct) was longest in G2, with a median of 851 min (DBD) and 944 min (DCD), compared to 615 min (DBD) and 612 min (DCD) in G1, and 435 min (DBD) and 571 min (DCD) in Direct to Tx (p < 0.001). Post-EVLP utilization rates were comparable across donor subtypes, with G1 at 66.6% (DBD) and 63.6% (DCD) and G2 at 67.1% (DBD) and 60.0% (DCD) (p > 0.05).

**Table 2:** Preservation Durations and Ischemia Times in EVLP and Direct Transplants

	**Direct Tx**		**G1**		**G2**		**p-value**							
	**DBD**	**DCD**	**DBD**	**DCD**	**DBD**	**DCD**	**DBD**				**DCD**			
	**(N=1306)**	**(N=269)**	**(N=298)**	**(N=278)**	**(N=49)**	**(N=75)**	**Direct vs G1 vs G2**	**Direct vs G1**	**Direct vs G2**	**G1 vs G2**	**Direct vs G1 vs G2**	**Direct vs G1**	**Direct vs G2**	**G1 vs G2**
CIT1 (min), median	360	501.5	285	265	377	445	< 0.001	< 0.001	1	< 0.001	< 0.001	< 0.001	< 0.001	< 0.001
EVLP time, (min) median	-	-	262	254	217.5	208				< 0.001				< 0.001
CIT2 (min), median	-	-	242	269	392.5	429				< 0.001				< 0.001
WIT (min), median	69	66	71	65	62	62	0.002	0.58	0.007	< 0.001	0.23	1	0.27	0.38
TIT (min) (CIT1+CIT2+WIT), median	435	571	615	612.5	851	944	< 0.001	< 0.001	< 0.001	< 0.001	< 0.001	1	< 0.001	< 0.001

Tx, transplant; G1, first-generation; G2, next-generation; DBD, donation after brain death; DCD, donation after circulatory death; CIT, cold ischemia time; WIT warm ischemia time; TIT, total ischemia time

Recipient characteristics are outlined in Table 3**.** Recipient age was higher in the G2 group, with a median of 65 years (IQR 59.5–71) compared to 61 years (IQR 52–67) in G1 and 59 years (IQR 49–66) in Direct to Tx. A higher proportion of bilateral lung transplants was performed in the Direct to Tx group (90.3%) compared to 75.3% in G1 and 82.3% in G2 (p < 0.05). The distribution of primary lung disease diagnoses varied among groups, with a higher proportion of ILD patients in the EVLP groups, and a significantly lower proportion of cystic fibrosis (CF) recipients in G2 (frequency in Direct to Tx vs G1 vs G2 10.5% vs 8.3% vs 1%, p = 0.002).

**Table 3:** Recipient Demographics and Transplant Characteristics by Group

**Factor**		**Direct to Tx**	**EVLP (G1)**	**EVLP (G2)**	**p-value**			
		**(N=1575)**	**(N=576)**	**(N=124)**	**Direct vs G1 vs G2**	**Direct vs G1**	**Direct vs G2**	**G1 vs G2**
Recipient age		59 (IQR 49–66)	61 (IQR 52–67)	65 (IQR 59.5–71)	<0.001	0.02	<0.001	<0.001
Recipient sex	Female	652 (41.4%)	210 (36.5%)	40 (32.3%)	0.026	0.13	0.17	1
	Male	923 (58.6%)	366 (63.5%)	84 (67.7%)				
Recipient BMI		24.1 (IQR 20.6–27.4)	24.8 (IQR 20.7–27.7)	24.6 (IQR 21.6–27.4)	0.135	0.19	0.96	1
Recipient diagnosis	ILD	684 (43.4%)	261 (45.3%)	76 (61.3%)	0.002	0.28	0.006	0.037
	COPD	387 (24.6%)	164 (28.5%)	24 (19.4%)				
	CF	166 (10.5%)	48 (8.3%)	1 (1%)				
	Re-Tx (CLAD/GF)	68 (4.3%)	24 (4.2%)	4 (3.2%)				
	PH	49 (3.1%)	16 (2.8%)	4 (3.2%)				
	Scleroderma	52 (3.3%)	14 (2.4%)	2 (1.6%)				
	Hypersensitivity Pneumonitis	34 (2.2%)	17 (3.0%)	4 (3.2%)				
	Others	135 (8.6%)	32 (5.6%)	9 (7.2%)				
Recipient pre-tx ECMO	Yes	104 (6.6%)	24 (4.2%)	2 (1.6%)	0.013	0.13	0.13	0.81
Tx type	Bilateral	1423 (90.3%)	434 (75.3%)	102 (82.3)	<0.001	<0.001	0.02	0.38
	Single	152 (9.7%)	142 (24.7)	22 (17.7%)				

Tx, transplant; G1, first-generation; G2, next-generation; IQR, interquartile range; BMI, body mass index; ILD, interstitial lung disease; COPD, chronic obstructive pulmonary disease; Re-Tx, re-transplant; CLAD, chronic lung allograft dysfunction; GF, graft failure; PH, pulmonary hypertension; ECMO, extracorporeal membrane oxygenation

Findings related to post-transplant clinical outcomes are presented in Table 4. Incidence of PGD3 at 72 h and median ventilation time were comparable across groups, with PGD3 occurring in15.4% of Direct to Tx, 15.9% of G1, and 9.7% of G2 (p = 0.205), and median ventilation times of 44 h in Direct to Tx, 48 h in G1, and 42 h in G2 (p = 0.448). Although ventilation time and PGD incidence did not differ significantly, the median ICU length of stay was one day longer in G2, with a median of 6 days (IQR 3–10.5) compared to 5 days (IQR 2–12.5) in Direct to Tx and 4 days (IQR 2–10) in G1 (p = 0.022). The total hospital length of stay remained comparable across groups, with 25 days in Direct to Tx, 22 days in G1, and 23 days in G2 (p = 0.016).

**Table 4:** Post-Transplant Clinical Outcomes Among EVLP and Direct Groups

**Endpoints**		**Direct to Tx**	**EVLP (G1)**	**EVLP (G2)**	**p-value**			
		**(N=1575)**	**(N=576)**	**(N=124)**	**Direct vs G1 vs G2**	**Direct vs G1**	**Direct vs G2**	**G1 vs G2**
PGD at 72 h	PGD3	196 (15.4%)	77 (15.9%)	12 (9.7%)	0.205	1	1	1
Time on extubation	<72 h	1024 (65.0%)	367(63.7%)	84 (67.7%)	0.671	1	1	1
	≧72 h	551 (35.0%)	209 (36.3%)	40 (32.3%)				
Ventilation time (h)		44 (IQR 24–120)	48 (IQR 24–96)	42 (IQR 19–107)	0.448	1	0.8	0.64
Post-Tx ECMO	Yes	87 (6.0%)	46 (8%)	6 (5%)	0.0895	0.14	1	0.92
ICU length of stay		5 days (IQR 2–12.5)	4 days (IQR 2–10)	6 days (IQR 3–10.5)	0.022	0.14	0.4	0.02
Hospital length of stay		25 days (IQR 17–44)	22 days (IQR 16–41)	23 days (IQR 16–35)	0.016	0.014	1	1

Tx, transplant; G1, first-generation; G2, next-generation; IQR, interquartile range; PGD, primary graft dysfunction; ECMO, extracorporeal membrane oxygenation; ICU, intensive care unit

**Figure 3:**
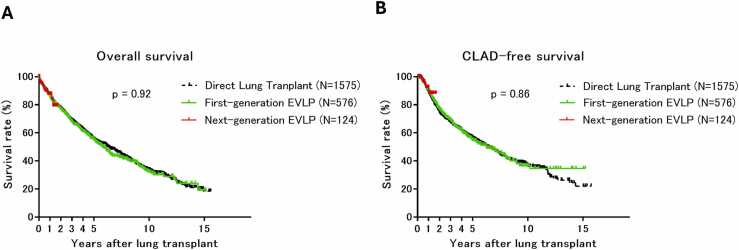
Overall Survival and CLAD-Free Survival. (A) Kaplan-Meier curves comparing overall survival among the three transplant groups: Direct lung transplant (Direct), first-generation EVLP (G1), and next-generation EVLP (G2). No statistically significant differences were observed between groups (log-rank p > 0.05). (B) Chronic lung allograft dysfunction (CLAD)-free survival across Direct, G1, and G2 groups. Survival rates were comparable, with no significant intergroup differences (log-rank p > 0.05).

Long-term survival outcomes demonstrated no statistically significant difference among groups. Overall survival (Figure 3A)and CLAD-free survival (Figure 3B) rates were comparable, with no significant difference among groups (p = 0.92 and 0.86, respectively). The median follow-up period for G2 recipients was 219 days (IQR 107–378.75).

## Discussion

This study focused on evaluating the clinical performance of the next-generation EVLP system, TorEx, by comparing post-transplant outcomes with those following conventional direct lung transplantation and first-generation EVLP. Despite a higher donor age (vs. G1), a greater proportion of DCD donors, and a lower P/F ratio (vs. Direct Tx) in next-generation EVLP, as well as older recipients and prolonged ischemia times, survival rates remained comparable among groups, and PGD incidence showed no significant difference. These findings suggest that procedural refinements in the TorEx EVLP system facilitated lung preservation without compromising transplant success. Moreover, the streamlined setup may improve accessibility in centers with limited EVLP experience, potentially enhancing donor lung utilization. This study provides the first evidence that next-generation EVLP can achieve outcomes comparable to both first-generation EVLP and conventional transplants, offering a novel solution to operational limitations noted in earlier research.

While this study compares outcomes between first- and next-generation EVLP, it is important to acknowledge that advancements in lung preservation and patient management have evolved over time. In recent years, donor lungs that were previously stored on ice are now preserved at 10°C, allowing for safer and prolonged cold ischemia times.^5^, ^6^ This shift in preservation strategy may have contributed to the ability of G2 to withstand extended ischemia durations without compromising transplant success. Additionally, lung transplantation trends have changed over the last decade, particularly among patients with diseases such as cystic fibrosis, which exemplifies broader shifts in clinical indications. The introduction of novel CF-modifying therapies has significantly reduced the number of CF patients requiring lung transplantation, altering recipient demographics and transplant indications.^7^ These factors highlight the importance of interpreting G1 and G2 comparisons within the context of evolving clinical practices and patient populations.

The TorEx EVLP system, designed as a plug-and-go model, streamlines lung preservation by simplifying setup and reducing personnel requirements. Compared to conventional EVLP systems, TorEx eliminates the need for assembling individual components such as pumps, oxygenators, reservoirs, and filters, allowing for a more efficient and standardized process. This innovation has led to a significant reduction in setup time, improving procedural feasibility for centers with limited EVLP experience. Additionally, the improved feasibility of real-time monitoring and perfusate exchange enables more accurate evaluation of graft function throughout the perfusion period. At Toronto General Hospital, the introduction of TorEx EVLP allowed for a reduction in required personnel from three to two, while maintaining post-transplant outcomes comparable to conventional EVLP approaches. These findings suggest that TorEx enhances operational efficiency without compromising clinical results. Since 2023, the system has been approved by Health Canada and is now recognized as a standard medical practice. This approval is expected to minimize technical challenges associated with traditional EVLP systems, further supporting its broader clinical integration. Furthermore, current utilization rates (conversion to transplant) is at 70%, substantially higher than published results of other EVLP platforms when using extended criteria lungs.^8^, ^9^, ^10^ However, cost considerations remain a factor, as the system requires specialized equipment and higher initial investment. Further studies are warranted to assess its long-term cost-effectiveness and overall clinical benefits.

The combination of 10°C static storage and intermittent EVLP has demonstrated the feasibility of preserving donor lungs for up to three days while maintaining excellent post-transplant function.^11^ This advancement significantly extends the traditional preservation window and could enhance organ transplantation logistics. The TorEx EVLP system’s cassette-based design provides additional practical benefits, allowing for seamless transitions between perfusion and cold storage. Unlike conventional EVLP systems, where lungs remain connected throughout, the cassette can be easily detached and transferred to refrigeration, simplifying long-term organ preservation. This approach may further improve donor lung utilization, particularly in cases where extended storage is required for logistical reasons. Future investigations should explore the clinical feasibility of integrating TorEx EVLP with cyclic EVLP protocols to refine long-term organ storage strategies and facilitate broader transplant accessibility.

This study had several limitations. As a single-center retrospective analysis, the findings may not be generalizable to all transplant programs. The shorter follow-up period for G2 recipients limited the assessment of long-term outcomes. Additionally, the learning curve for the new device, while not systematically analyzed, may have introduced variability in early results. Lastly, the groups compared in this study were established during different time periods and clinical settings, and therefore may reflect temporal differences in donor and recipient backgrounds. Accordingly, careful interpretation of intergroup comparisons is required, taking into account changes in patient characteristics and clinical settings over time.

In conclusion, the next-generation TorEx EVLP system demonstrated comparable short- and intermediate-term post-transplant recipient outcomes to conventional transplants without EVLP and to lung transplants following first-generation EVLP, while expanding the use of extended-criteria donor lungs. This innovation offers a practical solution to logistical challenges posed by traditional EVLP systems, with significant potential to increase donor lung availability and accessibility. Further multicenter prospective studies with longer follow-up periods are required to validate these findings and explore the full clinical impact of next-generation EVLP systems.

## Declaration of Competing Interest

The authors declare the following financial interests/personal relationships which may be considered as potential competing interests: A.A.: Employee of Traferox Technologies, J.C.Y.: Scientific Advisor to Traferox Technologies, T.W.: Consultant to Lung Bioengineering, shareholder of Traferox Technologies, S.K.: Consultant to Lung Bioengineering, shareholder and CMO of Traferox Technologies, M.C.: Consultant to Lung Bioengineering, shareholder and CSO of Traferox Technologies.
